# First Case of Patient With Two Homozygous Mutations in *MYD88* and *CARD9* Genes Presenting With Pyogenic Bacterial Infections, Elevated IgE, and Persistent EBV Viremia

**DOI:** 10.3389/fimmu.2019.00130

**Published:** 2019-02-14

**Authors:** Maria Chiriaco, Gigliola Di Matteo, Francesca Conti, Davide Petricone, Maia De Luca, Silvia Di Cesare, Cristina Cifaldi, Rita De Vito, Matteo Zoccolillo, Jessica Serafinelli, Noemi Poerio, Maurizio Fraziano, Immacolata Brigida, Fabio Cardinale, Paolo Rossi, Alessandro Aiuti, Caterina Cancrini, Andrea Finocchi

**Affiliations:** ^1^University Department of Pediatrics, Unit of Immune and Infectious Diseases, Childrens' Hospital Bambino Gesù, Rome, Italy; ^2^Department of Systems Medicine, University of Rome Tor Vergata, Rome, Italy; ^3^Histopathology Unit, Bambino Gesù Children's Hospital-Research Institute, Rome, Italy; ^4^San Raffaele Telethon Institute for Gene Therapy (SR-TIGET), IRCCS San Raffaele Scientific Institute, Milan, Italy; ^5^Department of Biology, University of Rome Tor Vergata Rome, Rome, Italy; ^6^Allergy, Immunology and Pediatric Pulmunology Unit, Policlinico di Bari Ospedale Giovanni XXIII, Bari, Italy; ^7^Pediatric Immunohematology, IRCCS San Raffaele Scientific Institute, Milan, Italy; ^8^Vita Salute San Raffaele University, Milan, Italy

**Keywords:** *MYD88*, *CARD9*, NGS, primary immune deficiency (PID), pyogenic infections

## Abstract

We described for the first time a female patient with the simultaneous presence of two homozygous mutations in *MYD88* and *CARD9* genes presenting with pyogenic bacterial infections, elevated IgE, and persistent EBV viremia. In addition to defective TLR/IL1R-signaling, we described novel functional alterations into the myeloid compartment. In particular, we demonstrated a defective production of reactive oxygen species exclusively in monocytes upon *E. coli* stimulation, the inability of immature mono-derived DCs (iDCs) to differentiate into mature DCs (mDCs) and the incapacity of mono-derived macrophages (MDMs) to resolve BCG infection *in vitro*. Our data do not provide any evidence for digenic inheritance in our patient, but rather for the association of two monogenic disorders. This case illustrates the importance of using next generation sequencing (NGS) to determine the most accurate and early diagnosis in atypical clinical and immunological phenotypes, and with particular concern in consanguineous families. Indeed, besides the increased susceptibility to recurrent invasive pyogenic bacterial infections due to MYD88 deficiency, the identification of *CARD9* mutations underline the risk of developing invasive fungal infections emphasizing the careful monitoring for the occurrence of fungal infection and the opportunity of long-term antifungal prophylaxis.

## Highlights

- Patient with atypical primary immunodeficiency
- Clinical manifestations: pyogenic bacterial infections, high IgE level, and persistent EBV viremia
- Next-generation sequencing reveals two homozygous mutations in *MYD88* and *CARD9* genes leading to complete absence of proteins
- Monocytes/macrophages function and DC differentiation were severely compromised
- NGS has a key role to determine the correct diagnosis in atypical primary immunodeficiency leading to reconsider the individualized treatment.

## Introduction

Primary immunodeficiency diseases (PIDs) are a heterogeneous group of disorders characterized by poor or absent function in one or more components of the immune system which predisposes affected individuals to recurrent and severe infections, autoimmunity, aberrant inflammation, atopy, lymphoproliferation, and malignancy. To date more than 350 different disorders have been genetically characterized and new disorders continually being recognized (1). PIDs are caused by defects in the adaptive immunity, T-cell, B-cell, or combined B and T cell immunodeficiencies, or innate immunity, phagocyte, NK cell, several classes of pattern recognition receptors (PRRs), and complement disorder (2).

The innate immune response represents the first line of defense against pathogens. Appropriate recognition of threats and induction of the downstream signaling cascades are essential steps in the removal of these organisms from the system. The failure of the innate system to identify pathogens, delays the induction of the immune response worsening outcomes of infection. Over the last two decades, several inborn errors affecting primarily components of innate immunity, conferring vulnerability to a narrow range of microorganisms, such as Mendelian susceptibility to mycobacterial disease (MSMD), herpes simplex 1 encephalitis (HSE), and monogenic susceptibility to invasive pneumococcal disease (IPD) have been identified (3).

Myeloid differentiation primary response protein 88 (MYD88) is a cytosolic adaptor protein recruited from most toll-like receptors (TLRs) and IL-1 receptors (IL-1Rs) to trigger the canonical pathway leading to NF-κB activation and inflammatory cytokine gene transcription. Patients with mutations in *MYD88* present a Mendelian predisposition to invasive (meningitis and septicaemia) and non-invasive bacterial infections caused principally by *S. pneumoniae, S. aureus* and, less frequently, by few Gram-negative bacteria. Typically, clinical and laboratory signs of inflammation (e.g., fever, elevated C-reactive protein level) in *MYD88* patients are weak or delayed even in case of severe infection (4–6). Moreover, an interesting feature is that contrary to the most of PID, in which patient's condition undergo a gradual deterioration over time, clinical status, and outcome in MYD88 deficient patients improve with age even in the absence of preventive measures, suggesting that the MyD88/TIR pathway becomes redundant once acquired immunity, is fully functional and can ensure protection.

Caspase-associated recruitment domain-containing protein 9 (CARD9) is a central regulator of innate immune response, acting as downstream regulator of Dectin-1, Dectin-2, Dectin-3, Mincle, and others involved in the recognition of fungal pathogene (7). Its deficiency causes a rare PID characterized by superficial and deep fungal infection in otherwise healthy individuals (HD). CARD9 deficiency cause defective cytokine production in response to fungal ligand, impaired T cell- dependent IL17 production and impaired neutrophil recruitment to infection sites and/or killing. Age at onset is heterogeneous and, although the most of reported patients showed an adult onset, fungal infections can occur at any age (8–11).

Here we report for the first time a female patient with inherited *MYD88* and *CARD9* deficiencies presenting with pyogenic bacterial infections, high level of IgE and persistent EBV viremia.

## Case Report

**Figure 1:**
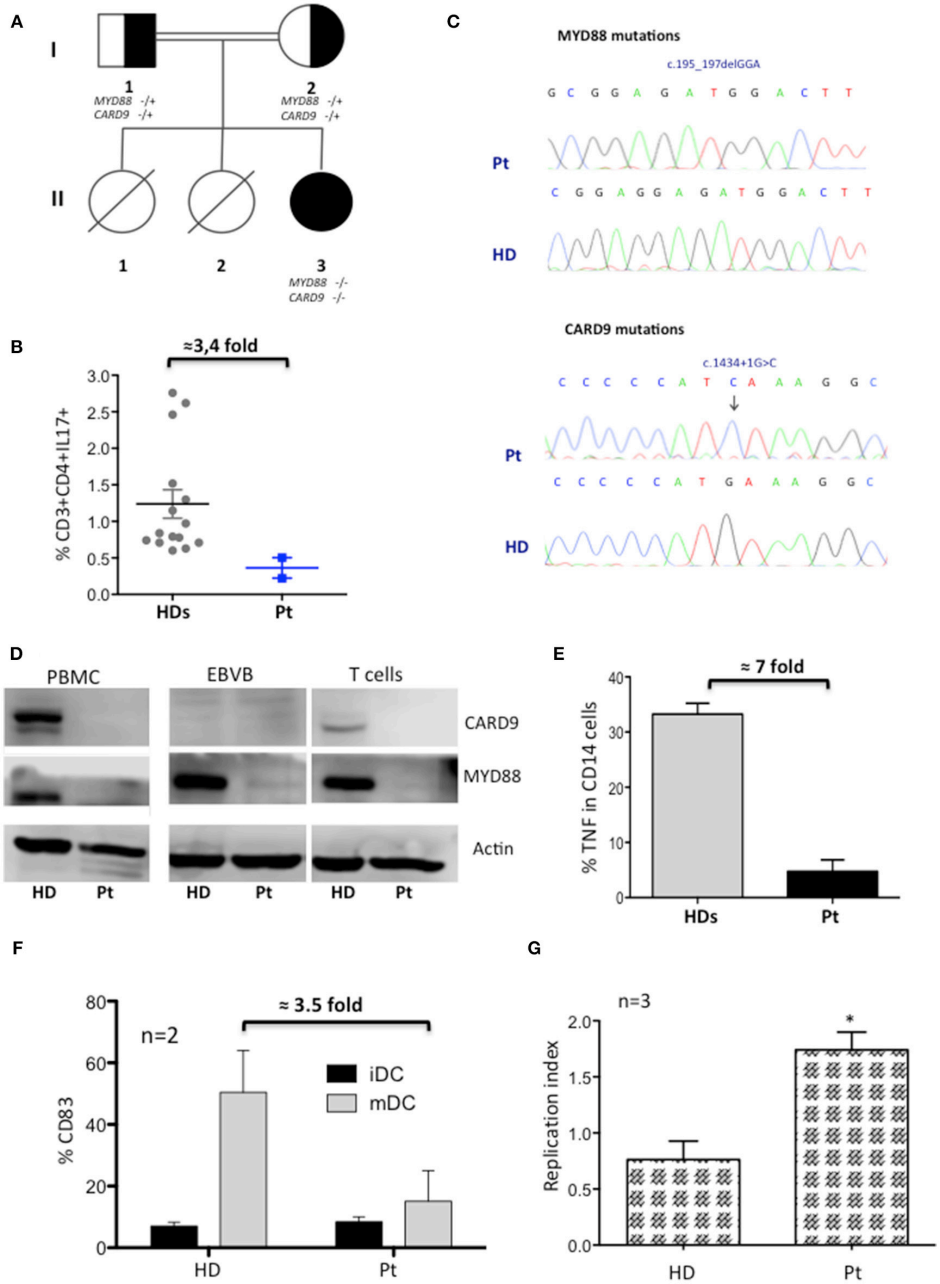
Characterization of MYD88-CARD9 deficiency patient: part 1. **(A)** Family pedigree showing proband (II3), carrier parents (I1, I2), and sisters (II1, II2). Genotype + and – indicate in the figure wild type and mutated, respectively. **(B)** Percentage of CD3+CD4+IL17+ cells after PMA stimulation (mean ± SEM of *n* = 2). **(C)** Sanger sequencing confirmed a homozygous in-frame deletion (c.195_197delGGA) in *MYD88* gene and a homozygous splice-donor mutation (c.1434+1G>C) in *CARD9* gene. **(D)** Western Blot of CARD9 and MYD88 proteins performed on PBMC, EBVB, and PHA derived T cell lines. **(E)** TNFα production by monocytes after LPS stimulation (mean ± SEM of *n* = 2). **(F)** Phenotypic analysis of iDC and mDC differentiated *in vitro*. Results indicate the mean percentage ± SD of CD83 maturation/activation markers in gated CD14-CD1a+HLA-DR+ subsets of iDC and mDC. **(G)** Capacity of MDM to kill (right panel) BCG (^*^P < 0.05).

A female infant patient was born from consanguineous parents with a family history suggestive for PID. Her sisters deceased, respectively, for meningitis and sepsis of unknown origin (Figure 1A). The umbilical cord fell at 30 days and she was fully immunized including measles, mumps, and rubella, without any complications. During the first year of life she suffered from recurrent respiratory infections and one episode of urinary tract infection due to *P. aeruginosa*. At the age of 2 years she was hospitalized for an intramuscular and deep abdominal abscess caused by *S. aureus*, without any signs of inflammation, which was successfully treated with a combined therapy of intravenous antibiotics and surgical drainage. Extensive immunological work up revealed a normal complete blood count (CBC), normal number of T-, B-, and NK-cells (including naïve and memory subsets, and CD4^+^CD31^+^), normal nitroblue tetrazolium (NBT) test, normal serum immunoglobulin levels and normal specific antibody response to *T. toxoid*, HBV, and HIB. We detected high levels of IgE (4,550 KU/L), mild eosinophilia (900/mcL), and non-protective response to pneumococcal vaccines. In spite of elevated circulating IgE levels, patient did not develop allergic symptoms and allergen-specific IgE was not detectable. During follow-up, despite co-trimoxazole prophylaxis, she developed urinary tract infection caused by *E. coli* and a relapsing necrotizing granulomatous lymphadenitis of left cervical lymph nodes, not responding to antibiotic therapy, and successfully treated with surgical removal. Moreover, the patient showed persistent EBV viremia in the blood with no evidences of organs damage. Further analysis revealed a normal frequency of Tregs and Tfh cells, and a normal level of INFγ produced by CD4^+^ and CD8^+^ T cells after PMA stimulation (data not shown). Patient showed lower frequency (3.4-fold decreased) of circulating Th17 with respect to HD (Pt 0.36 ± 0.14 vs. HDs 1.23 ± 0.19%) (Figure 1B). Moreover, a mild decreased level of memory B cells with impaired B cells responsiveness to CpG-TLR9 stimulation was observed (data not shown).

Although the clinical phenotype was not typical of the hyper IgE syndromes (HIES), family history, elevated levels of IgE, eosinophilia, and decreased Th17 cells led us to exclude by Sanger sequencing genetic defects associated with HIES (*STAT3, DOCK8*, and *TYK2*). Moreover, IRAK4 PID variants were excluded considering the absence of fever and of a significant increase of inflammatory markers during severe infections. HaloPlex targeted sequencing panel, including 630 causative and candidate PID genes (Cifaldi et al., manuscript submitted) identified two homozygous mutations in two different genes (Figures 1C,D and Supplementary Table 1). A known pathogenic in-frame deletion c.195_197delGGA (AF of 8.13180e-06_*1000 Genomes*) in exon1 of the *MYD88* gene leading to absent protein expression resulting in a loss-of-function mutation (4), and a homozygous splice-donor variant c.1434+1G>C (AF of G = 0.0008/4_*1000 Genomes*) in the *CARD9* gene, with absent protein, previously described as heterozygous variant (11). Sanger sequencing confirmed the mutations and both parents resulted heterozygous (data not shown).

Since both mutated proteins caused impaired innate immunity we deeply investigated the myeloid compartment. Patient's monocytes showed a marked reduction of TNFα-production (7-fold decreased) after LPS-stimulation (Figure 1E), although PB-monocytes (classical, intermediate, and non-classical), and major subtypes of dendritic cells (mDC1, mDC2, and pDC) were normally represented (data not shown). Moreover, immature monocytes-derived DC had a defective ability to become mature/activated-DC (mDC) (3.5-fold decrease) (Figure 1F), while maintaining their phagocytic capacity (data not shown). Additionally, patient's monocytes-derived macrophages (MDMs) failed to kill Bacillus Calmette–Guérin (BCG) *in vitro* (Figure 1G) after engulfment (12–14).

**Figure 2:**
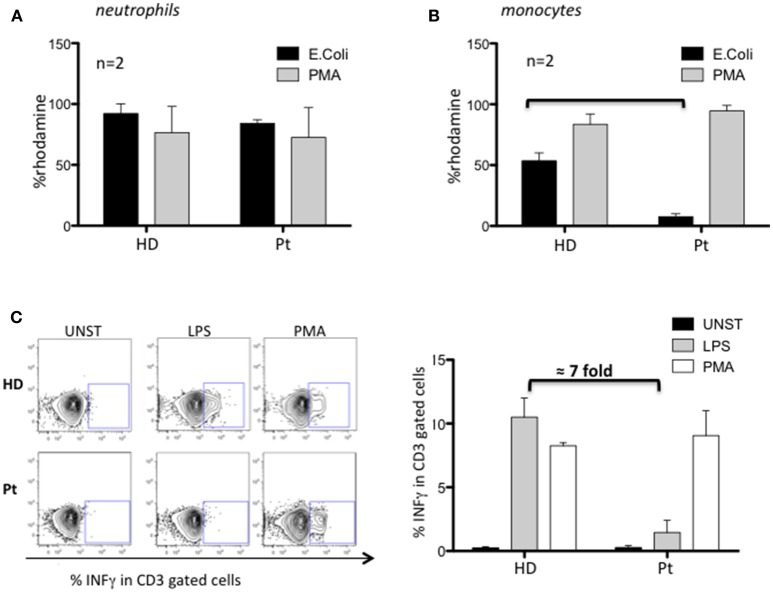
Characterization of MYD88-CARD9 deficiency patient: part 2. **(A,B)** NADPH oxidase activity evaluated by dihydrorhodamine (DHR) assay in patient's neutrophils and monocytes stimulated with PMA or *E. coli*. The production of ROS is determined by the oxidation of DHR into the fluorescent rhodamine. **(C)** INFγ production by patient's CD3 cells stimulated with PMA or LPS/monocytes. *Left panel* shows representative plots and *right panel* shows mean ± SEM of *n* = 2.

Next, we investigated the nicotinamide adenine dinucleotide phosphate (NADPH) oxidase activity by dihydrorhodamine (DHR) flow cytometric assay using PMA and opsonized *E. coli* to stimulate neutrophils and monocytes. Neutrophils from our patient performed a normal respiratory burst (Figure 2A), in agreement with what previously observed in MYD88 and CARD9 deficient patients. Conversely, we found an impaired capacity to produce ROS in patient's monocytes selectively stimulated with *E. coli* (7-fold decreased respect to HD) (Figure 2B). Interestingly, these results showed the impaired contact signal/trigger to external stimuli by monocytes, which could in part explain, the susceptibility to urinary tract infection. To confirm this inability, we tested the capacity of monocytes to stimulate T cells via LPS to produce INFγ and found a defective LPS/monocytes-triggered INFγ production by patient's CD3 cells (7-fold decreased) as compared to HD (Figure 2C).

## Discussion

Here we describe a patient with a phenotype of recurrent respiratory and deep tissue bacterial infections with granuloma inflammation in whom two pathogenic homozygous mutations in *MYD88* and *CARD9* genes were identified by NGS. In agreement with MYD88- and CARD9-deficiencies, we showed a defective TLR/IL1R-signaling leading to a severe decrease of TNFα and IL17 pro-inflammatory cytokines production, that compromise neutrophils recruitment to the site of acute inflammation (Supplementary Figure 1) and the capacity of the immune system to definitely resolve bacterial infections (15). The patient has a severely compromised myeloid compartment. Indeed, peripheral monocytes were unable to differentiate into mDCs, and MDMs cells do not resolve *in vitro* BCG infection. Although NADPH-oxidase respiratory burst is normally functioning after PMA-stimulation, monocytes are severely unable to produce ROS in response to *E. coli*. These data highlight the inability of patient monocytes to receive and process external signals and could explain the development of *P. aeruginosa, S. aureu*s, and *E. coli* infections in our patient. Additionally, we investigated if *MYD88*-*CARD9* mutations hinder the development of an appropriate T cell response. We found a marked defect (7-fold decrease) of INFγ production by CD3 cells after LPS-monocytes stimulation revealing a compromised cooperation between monocytes and T cells.

High level of serum IgE, family history, recurrent bacterial infections, and low level of circulating Th17 initially suggested us a diagnosis of HIES or IRAK4 deficiency shortly refuted by molecular diagnosis of CARD9 and MYD88 deficiencies. Frans et al. (16) similarly to our patient, described an adult female patient who was at first phenotypically diagnosed with HIES because of severe bacterial and fungal infections, and elevated serum IgE levels, but finally defined as suffering from IRAK-4 deficiency. These findings highlight the importance of using NGS to identify the genetic cause of the disease in a patient presenting with atypical clinical and immunological phenotype. Considering IRAK-4 deficiency as a phenocopy of MYD88 deficiency, although both CARD9 (10) and MYD88 (6) deficiency showed moderately increased level of IgE compared to the very high levels found in HIES patients (17), we believe that MYD88 plays a crucial role in determining the patient's phenotype.

We further demonstrate that MDMs were unable to resolve *in vitro* BCG infection. Interestingly, MYD88 deficiency mice showed an increased susceptibility and mortality to the experimental infection with three Mycobacteria species (*M. avium, M. bovis*, and *M. tubercolosis*) and Mycobacterium avium infection has been reported in one IRAK4-deficient patient (18) thus suggesting a possible role for MYD88/IRAK4 signaling axis in protective immunity to Mycobacteria.

**Figure 3:**
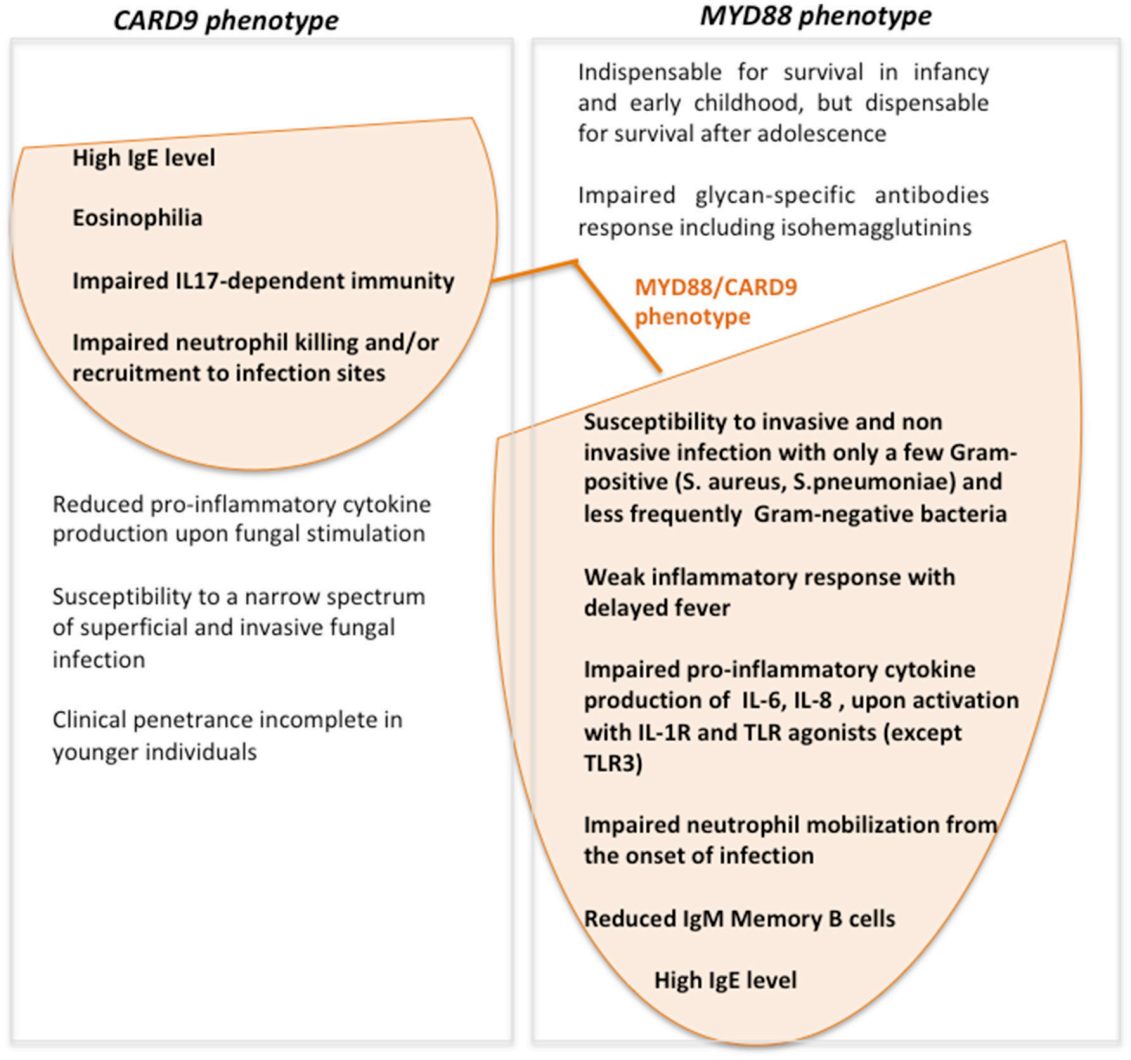
Venn diagram showing MYD88- and CARD9-deficiency features and clinical/laboratory data of our patient with the simultaneous presence of *MYD88* and *CARD9* mutations.

Interestingly our patient had a persistent-asymptomatic EBV viremia commonly reported in several PID patients but not typically associated with CARD9- or MYD88-deficiency (19). The impaired TLR2/MYD88 axis observed in our patients by the absence of Myd88 protein could induce a defective cytokines production that allows EBV to establish persistent infections evading immune surveillance (20, 21). The role of Card9 protein during EBV infection still remains unknown. This condition could be depend by the simultaneous presence of both defects that may create the proper context to allow the EBV to replicate, or by the presence of any other gene unknown variants that could increase the susceptibility to EBV. Despite the persistent asymptomatic EBV viremia represents the only new feature as compared to MYD88- and CARD9- deficiency phenotype, we do not feel to define our patient as affected by digenic PID (22, 23), but rather with two monogenic disorders. Accordingly to the patient's age the predominant clinical phenotype is the one associated with MYD88-deficiency (Figure 3). The age at onset of fungal infection, characteristic for *CARD9*-deficiency, is highly heterogeneous and could arise from childhood to adulthood (10), with incomplete clinical penetrance in younger individuals (24).

## Concluding Remarks

We described for the first time a patient with the simultaneous presence of two homozygous variant mutations in *MYD88* and *CARD9* genes presenting with pyogenic bacterial infections, elevated IgE and persistent EBV viremia. In addition to the defective TLR/IL1R-signaling, we demonstrated a selective impaired monocytes/macrophages function and DC differentiation. This study shows the difficult path to perform a definitive diagnosis if patient's phenotype is atypical. Although NGS technologies have a key role to discover mutated genes, a continuous critical comparison with the patient clinical manifestations is needed to improve clinical management. Indeed, besides the increased susceptibility to recurrent invasive pyogenic bacterial infections due to MYD88-deficiency, the identification of *CARD9* mutations underline the risk of developing invasive fungal infections emphasizing the importance of careful monitoring for the occurrence of fungal infection and the opportunity of long-term antifungal prophylaxis.

## Ethics Statement

All procedures performed in the study were in accordance with the ethical standards of the institutional research committee and with the 1964 Helsinki declaration. Written informed consent, following standard ethical procedures with approval of the Children's Hospital Bambino Gesù Ethical Committee, was obtained from parents of patient.

## Author Contributions

MC designed and performed experiments, analyzed the data, and wrote the paper. GD performed NGS and contributed to revision the manuscript. FrC and FaC contributed to therapeutic approach and wrote the paper. DP and MZ performed the bioinformatics analyses. SD, CrC, NP, MF, IB, and RD performed laboratory studies and contributed to data interpretation. MD, JS, and PR have been important part in complicated diagnostic and therapeutic course. AA supervise the project and contributed to interpretation and revision of the manuscript. CaC and AF supervised the project and wrote the manuscript.

### Conflict of Interest Statement

The authors declare that the research was conducted in the absence of any commercial or financial relationships that could be construed as a potential conflict of interest.

## Abbreviations

AF: allelic frequency
BCG: bacillus calmette-guerin
CARD9: caspase-associated recruitment domain-containing protein 9
CBC: complete blood count
EBV: epstein-barr virus
IL-1Rs: IL1 receptors
IPD: invasive pneumococcal disease
HBV: hepatitis B virus
HD: healthy donor
HIB: Haemophilus Influenzae B
HIES: hyper IgE syndrome
HSE: herpes simplex virus 1 encephalitis
MDCs: mono-derived dendritic cells
MDMs: mono-derived macrophages
MSMD: mendelian susceptibility to mycobacterial disease
MYD88: myeloid differentiation primary response protein 88
NADPH: nicotinamide adenine dinucleotide phosphate
NGS: next-generation sequencing
NF-κB: nuclear factor kappa-light-chain-enhancer of activated B cells
PB: peripheral blood
PCR: polymerase chain reaction
PID: primary Immunedeficiency
PRRs: pattern recognition receptors
ROS: reactive oxygen species
Tfh: T follicular helper cells
TLRs: Toll-like receptors
Tregs: T regulatory cells.

## References

[1] Picard C Bobby Gaspar H Al-Herz W Bousfiha A Casanova JL Chatila T. Primary immunodeficiency diseases committee report on inborn errors of immunity. J Clin Immunol. (2018) 38:96–128. 10.1007/s10875-017-0464-9 29226302 PMC5742601

[2] McCusker C Upton J Warrington R. Primary immunodeficiency. Allergy Asthma Clin Immunol. (2018) 14 (Suppl. 2):61. 10.1186/s13223-018-0290-5 30275850 PMC6157160

[3] Bucciol G Moens L Bosch B Bossuyt X Casanova JL Puel A. Lessons learned from the study of human inborn errors of innate immunity. J Allergy Clin Immunol. (2018). 10.1016/j.jaci.2018.07.013. [Epub ahead of print]. 30075154 PMC6358521

[4] Alsina L Israelsson E Altman MC Dang KK Ghandil P Israel L A narrow repertoire of transcriptional modules responsive to pyogenic bacteria is impaired in patients carrying loss-of-function mutations in MYD88 or IRAK4. Nat Immunol. (2014) 15:1134–42. 10.1038/ni.3028 25344726 PMC4281021

[5] Picard C Casanova JL Puel A Infectious diseases in patients with IRAK-4, MyD88, NEMO, or IκBα deficiency. Clin Microbiol Rev. (2011) 24:490–7. 10.1128/CMR.00001-11 21734245 PMC3131061

[6] Von Bernuth H Picard C Jin Z Pankla R Xiao H Ku CL. Pyogenic bacterial infections in humans with MyD88 deficiency. Science (2008) 321:691–6. 10.1126/science.1158298 18669862 PMC2688396

[7] Drummond RA Franco LM Lionakis MS. Human CARD9: a critical molecule of fungal immune surveillance. Front Immunol. (2018) 9:1836. 10.3389/fimmu.2018.01836 30127791 PMC6088205

[8] Glocker EO Hennigs A Nabavi M Schäffer AA Woellner C Salzer U. A homozygous CARD9 mutation in a family with susceptibility to fungal infections. N Engl J Med. (2009) 361: 1727–35. 10.1056/NEJMoa0810719 19864672 PMC2793117

[9] Lanternier F Mahdaviani SA Barbati E Chaussade H Koumar Y Levy R. Inherited CARD9 deficiency in otherwise healthy children and adults with Candida species-induced meningoencephalitis, colitis, or both. J Allergy Clin Immunol. (2015) 135:1558–68.e2. 10.1016/j.jaci.2014.12.1930 25702837 PMC4831587

[10] Lanternier F Pathan S Vincent QB Liu L Cypowyj S Prando C. Deep dermatophytosis and inherited CARD9 deficiency. N Engl Med. (2013) 369:1704–14. 10.1056/NEJMoa1208487 24131138 PMC4084693

[11] Drummond RA Lionakis MS. Mechanistic insights into the role of C-type lectin receptor/CARD9 signaling in human antifungal immunity. Front Cell Infect Microbiol. (2016) 6:39. 10.3389/fcimb.2016.00039 27092298 PMC4820464

[12] Szymanski EP Leung JM Fowler CJ Haney C Hsu AP Chen F. Pulmonary nontuberculous mycobacterial infection. a multisystem, multigenic disease. Am J Respir Crit Care Med. (2015) 192:618–28. 10.1164/rccm.201502-0387OC 26038974 PMC4595692

[13] Berod L Stüve P Swallow M Arnold-Schrauf C Kruse F Gentilini MV. MyD88 signalling in myeloid cells is sufficient to prevent chronic mycobacterial infection. Eur J Immunol. (2014) 44:1399–409. 10.1002/eji.201344039 24435955

[14] Dorhoi A Desel C Yeremeev V Pradl L Brinkmann V Mollenkopf HJ. The adaptor molecule CARD9 is essential for tuberculosis control. J Exp Med. (2010) 207:777–92. 10.1084/jem.20090067 20351059 PMC2856020

[15] Rieber N Gazendam RP Freeman AF Hsu AP Collar AL Sugui JA. Extrapulmonary *Aspergillus* infection in patients with CARD9 deficiency. JCI Insight (2016) 1:e89890. 10.1172/jci.insight.89890 27777981 PMC5070961

[16] Frans G Moens L Schrijvers R Wuyts G Bouckaert B Schaballie H. PID in disguise: molecular diagnosis of IRAK-4 deficiency in an adult previously misdiagnosed with autosomal dominant hyper IgE syndrome. Clin Immunol. (2015) 35:739–44. 10.1007/s10875-015-0205-x 26472314

[17] Al-Shaikhly T Ochs HD. Hyper IgE syndromes: clinical and molecular characteristics. Immunol Cell Biol. (2018). 10.1111/imcb.12209. [Epub ahead of print]. 30264496

[18] Picard C von Bernuth H Ghandil P Chrabieh M Levy O Arkwright PD. Clinical features and outcome of patients with IRAK-4 and MyD88 deficiency. Medicine (2010) 89:403–25. 10.1097/MD.0b013e3181fd8ec3 21057262 PMC3103888

[19] Cohen JI. Primary immunodeficiencies associated with EBV disease. Curr Top Microbiol Immunol. (2015) 390 (Pt 1):241–65. 10.1007/978-3-319-22822-8_10 26424649 PMC6349415

[20] Ariza ME Glaser R Kaumaya PT Jones C Williams MV. The EBV-encoded dUTPase activates NF-kappa B through the TLR2 and MyD88-dependent signaling pathway. J Immunol. (2009) 182:851–9. 10.4049/jimmunol.182.2.851 19124728 PMC12892303

[21] Chijioke O Azzi T Nadal D Münz C. Innate immune responses against Epstein Barr virus infection. J Leukoc Biol. (2013) 94:1185–90. 10.1189/jlb.0313173 23812328 PMC3828602

[22] Ameratunga R Woon ST Bryant VL Steele R Slade C Leung EY. Clinical implications of digenic inheritance and epistasis in primary immunodeficiency disorders. Front Immunol. (2018) 8:1965. 10.3389/fimmu.2017.01965 29434582 PMC5790765

[23] Ehlayel M de Beaucoudrey L Fike F Nahas SA Feinberg J Casanova JL. Simultaneous presentation of 2 rare hereditary immunodeficiencies: IL-12 receptor beta1 deficiency and ataxia-telangiectasia. J Allergy Clin Immunol. (2008) 122:1217–9. 10.1016/j.jaci.2008.07.005 18718650

[24] Corvilain E Casanova JL Puel A. Inherited CARD9 deficiency: invasive disease caused by ascomycete fungi in previously healthy children and adults. J Clin Immunol. (2018) 38:656–93. 10.1007/s10875-018-0539-2 30136218 PMC6157734

