# Supplementary material for: First Case of Patient With Two Homozygous Mutations in MYD88 and CARD9 Genes Presenting With Pyogenic Bacterial Infections, Elevated IgE, and Persistent EBV Viremia
Source: Front Immunol. 2019 Feb 14;10:130. doi: 10.3389/fimmu.2019.00130 (PMC6383679; doi:10.3389/fimmu.2019.00130)
Supplement: Supplementary file 1 [file Data_Sheet_1.docx]

Supplementary Material

**First case of patient with two homozygous mutations in *MYD88* and *CARD9* genes presenting with pyogenic bacterial infections, elevated IgE and persistent EBV viremia**

Maria Chiriaco, PhD^1*^, Gigliola Di Matteo, PhD^2^, Francesca Conti, MD, PhD^1^, Davide Petricone, PhD^2^, Maia De Luca, MD^1^, Silvia Di Cesare, MSc^2^, Cristina Cifaldi, PhD^1^, Rita De Vito^3^, Matteo Zoccolillo, MSc^4^, Jessica Serafinelli, MD^1^, Noemi Poerio, MSc^5^, Maurizio Fraziano, MD, PhD^5^, Immacolata Brigida, PhD^4^, ^6^Fabio Cardinale, MD, Paolo Rossi MD, PhD^1,2^, Alessandro Aiuti, MD, PhD^4,7,8^, Caterina Cancrini, MD, PhD ^1,2§^ and Andrea Finocchi, MD, PhD^1,2 §*^

*** Correspondence:**

-Maria Chiriaco, MSc,PhD.

Email: [mary.chiriaco@gmail.com](mailto:mary.chiriaco@gmail.com)

-Andrea Finocchi, MD,PhD.

Email: [andrea.finocchi@uniroma2.it](mailto:andrea.finocchi@uniroma2.it)

# Supplementary Data

**Methods Sections**

*Sanger Sequencing:* HaloPlex targeted sequencing panel including 630 genes causing PID was performed on 200ng DNA isolated from peripheral blood. The resulting variants were filtered using a home-made database (Table E1).

Sanger sequencing was used to confirm the causal mutations. PCR reactions were: 95°C for 5 minutes, followed by 30-40 cycles at 95°C for 30 seconds, annealing at 60°C for 30 seconds, 72°C for 30 seconds, 72°C for 5 minutes. Primers used: CARD9, for: GCCTCCTGGAGCAATGAA and rev: GTGGGCAGAGACCTTGTG; MYD88, for: CTGCTGAGCTCCAAAGATGA and rev: TCCTCCAGTACCTGGATGTAG.

*Immunoblot analysis:* Human PBMCs were isolated by density-gradient centrifugation with Ficoll-Paque PLUS (GE Healthcare). CD3+ T cells and CD3- cells isolated by CD3 MicroBeads positive selection (Milthenyi) were washed in cold PBS and were immediately lysed in JS1X buffer (50 mM Tris/HCl ph 8, 150 mM NaCl, 1.5 mM MgCl2, 5 mM EGTA, 1% Triton-X, 10% glycerol, 1 mM PMSF, aprotinin 1 mg/ml, leupeptin 1 mg/ml, pepstatin 1 mg/ml, 1 mM DTT and phosphatase inhibitor cocktails (SIGMA) for 20 minutes on ice. Then they clarified by centrifugation at 1600 rpm for 10 min at 4°C. Total cell lysate was size-fractionated by SDS-PAGE gel (14%) and then transferred to nitrocellulose membrane (Protran by Schleicher & Schuell-Bioscience, Dassel, Germany). Membranes were blocked in 5% BSA for 1 h at room temperature and then incubated at T=room with CARD9 antibody (1h 1:500, Cell Signaling), B-Actin (1h, 1:3000, SIGMA), MYD88 (1h 1:500, Cell Signaling) and then with secondary goat-anti rabbit IgG (Cell Signaling) or goat-anti mouse IgG (Cell Signaling) for 1h/T room. Proteins were visualized by SuperSignal WestDura Extended Duration Substrate (Thermo Scientific).

*Intra-cellular staining*: *Th17*, PBMC were stimulated with PMA o.n. (100ng/ml; Sigma Aldrich) and ionomycin (1ug/ml; Sigma Aldrich). Brefeldin A (10ug/ul; Sigma Aldrich) was added after the beginning of the incubation. Then cells were stained with CD4-APC for 20’ at T room and then fixed and permeabilized. Then cells were washed, stained with IL17-FITC (BD) 20’ T=room and finally analysed by FACS. *TNFα,* PBMC were cultured with LPS (1mg/ml, SIGMA) for 6h at 37°C in presence of Brefeldin A. Then cells were stained with CD11b-APC (surface) and TNFa-PE (intra-cellular) 20’ T=room and finally analysed by FACS.

*INFγ−*, PBMC were cultured with LPS (1mg/ml, SIGMA) and PMA (1mg/ml) for 6h at 37°C in presence of Brefeldin A. Then cells were stained with CD3-PERCP (surface) and INFγ-FITC (intra-cellular) for 20’ T=room and finally analysed by FACS.

*ROS production investigation*: NADPH-oxidase activity was determined by PhagoBurst Test following manufacturer’s instructions (OrpegenPharma, Germany). Briefly, 100 ul of blood/for sample was filled with 20 ul of opsonized bacteria E. coli and 20 μL of PMA, a strong non-receptor activator (high control). All samples were incubated for 10 min at 37.0 °C in a water bath, dihydrorhodamine (DHR) 123 as a fluorogenic substrate was added and incubated again in the same conditions. The oxidative burst occurred with the production of reactive oxygen substrates (ROS) (superoxide anion, hydrogen peroxide) in granulocytes stimulated. In ROS-stimulated granulocytes, nonfluorescent DHR 123 underwent conversion to fluorescent rhodamine (R) 123 registered in the flow cytometer FACS Canto II.

*Monocytes-derived Dendritic Cells (moDC) differentiation:* CD14+ monocytes were isolated from PBMCs by positive selection using anti-CD14-conjugated magnetic MicroBeads (MACS-Miltenyi), according to manifacturer’s instruction. DCs were differentiated by culturing CD14+ cells with rhGM-CSF (100 ng/ml; R & D Systems) and rh IL-4 (10 ng/ml; R & D Systems) for 5 days in RPMI-1640 containing 10% FBS with Penicillin/Streptomicin/Glutamin (GIBCO). After, DCs were cultured for an additional 2 days w/o LPS (1ug/ml SIGMA) in order to induce mature (mDC) and immature (iDC) dendritic cells respectively. The purity and maturation state of DC were checked by FACS analysis to determine expression of CD14, CD1a, HLA-DR, CD83 and CD86 (BD).

*Macrophages (MDM) differentiation and BCG assay:* CD14+ cells were plated for 2 h in RPMI without FBS and P/S/G. After, cells were washed and cultured in complete RPMI (FBS/P/S/G) with M-CSF (50ng/ml; Miltenyi Biotec) for 7 days in order to differentiate macrophages. Differentiated cells (CD206+CD14+) were plated in RPMI+FBS+G without antibiotics and infected with mycoacterium bovis BCG Pasteur strain (TMC1011) at MOI 5 for 3 days. Intracellular BCG growth was monitored by CFU assay (Poerio et al., 2015, Poerio et al., 2017) after 3-hour and at day 3 post-infection. In particular bacilli obtained from cells after saponin treatment were plated in triplicate. BCG replication index represented the ratio between the CFU (BCG) obtained after 3 day post-infection and CFU (BCG) obtained after 3 hours of exposure to BCG.BCG phagocytosis index was evaluated as the ratio between the CFU (BCG) obtained after 3 hours of infection and the CFU (BCG) obtained from inoculum.

# Supplementary Figures and Tables

## Supplementary Figures


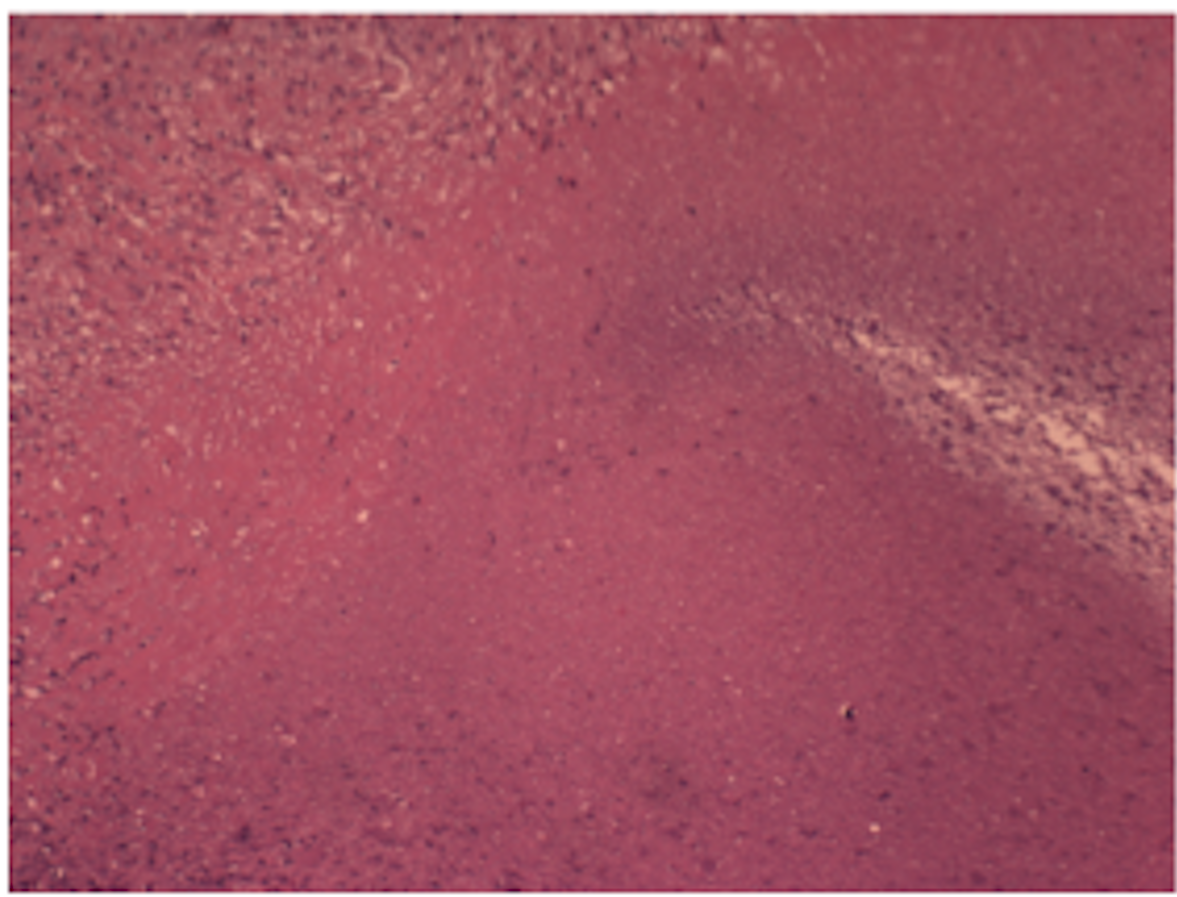


**Supplementary Figure 1.** Histological picture of the lymph nodes revealing granulomatous lesion with central coagulative necrosis without neutrophils infiltration

**Table 1.** **HaloPlex targeted sequencing summary**. BAM files were analyzed by a Perl script with SamTools module and filtered through standard mapping quality (MAPQ=30 with error rate: 0.001) and base quality (BQ>=10) to obtain a VCN file good quality variants. VCF ﬁles were imported into home-made database considering patient ID, sequencing platform, target panel information and variant information obtained by wANNOVAR or ANNOVER. All this information were used as variant ﬁltering for each sample.
